# Low Birthweight (LBW) and Neonatal Hyperbilirubinemia (NNH) in an Indian Cohort: Association of Homocysteine, Its Metabolic Pathway Genes and Micronutrients as Risk Factors

**DOI:** 10.1371/journal.pone.0071587

**Published:** 2013-08-06

**Authors:** Krishna Kishore Sukla, Pankaj Kumar Tiwari, Ashok Kumar, Rajiva Raman

**Affiliations:** 1 Cytogenetics Laboratory, Department of Zoology, Institute of Medical Sciences, Banaras Hindu University, Varanasi, Uttar Pradesh, India; 2 Centre for Genetic Disorders, Institute of Medical Sciences, Banaras Hindu University, Varanasi, Uttar Pradesh, India; 3 Department of Pediatrics, Institute of Medical Sciences, Banaras Hindu University, Varanasi, Uttar Pradesh, India; University of Montreal, Canada

## Abstract

**Background & Aims:**

Indian subcontinent has the highest child mortality rates along with a very high frequency of low birthweight (LBW). Folate and vitamin B12 (Vit-B12) are necessary during foetal development and their deficiency prevalence in Indians is very high. The objective of the present paper is to assess whether foetal homocysteine (Hcy)/folate metabolic pathway genes, their cofactors and homocysteine level independently (or collectively) predispose children to Low birth weight.

**Methods:**

Cord blood was collected for the study. Frequency of 5 SNPs in 4-Hcy-pathway genes, and levels of Hcy, Vit-B12 and folate were evaluated.

**Results:**

Of the 421 newborns recruited for the study, 38% showed low birth weight (<2.5kg) and 16% were preterm babies. 101 neonates developed neonatal hyperbilirubinemia (NNH). High prevalence of Vit-B12 (65%) and folate (27%) deficiency was observed in newborns along with hyperhomocystinemia (hypHcy-25%). Preterm delivery, micronutrient deficiency, hypHcy and MTHFR 677T SNP are associated as risk factor while G allele of TCN2 C776G is protective against LBW. MTHFR 677T allele and folate deficiency are also independent risk factors for NNH.

**Conclusion:**

We record the highest incidence of Vit-B12, folate deficiency and elevated Hcy levels, of all the studies so far reported on neonates. These together with MTHFR 677T are potential risk factors for LBW. Association of impaired folate/Hcy metabolism with NNH is reported for the first time and the possible way of interaction is discussed. It appears that proper nutritional management during pregnancy would reduce the risk of complex clinical outcomes.

## Introduction

World Bank report 2011 shows that globally the infant (5.7%) and child mortality (6.7%) rate in India [1] is among the highest and particularly more severe in the eastern Indian states of Uttar Pradesh and Bihar [2]. Out of ~9 million cases of child mortality recorded worldwide in 2008, 40% belong to Indian subcontinent (India-27%; Pakistan-7%, Bangladesh -4% and 2% from Afghanistan) [3]. Neonatal deaths (<27days) in India are estimated to account for 54% of deaths in children younger than 5 years [3]. The known direct causes of neonatal death are preterm birth (28%), severe infections (26%), and asphyxia (23%). Low birth weight (<2.5kg) is also an important indirect cause of death in early life [3]. India is considered the world capital for low birthweight (LBW) [4]. Newborns with LBW along with premature delivery are associated with clinical ailments related to growth and neuronal development, rendering the children susceptible to early mortality [5]. With high rates of child mortality and stunted growth (52 million children <5years), India’s burden of child health and nutrition is likely to be greater than in any other country [6].

Several studies have shown that accumulation of the thiol amino acid, Homocysteine (Hyperhomocysteinemia; hypHcy), either due to mutations in the metabolism pathway genes (viz., *MTHFR, CBS, MTRR*, and *SLC19A1*) or deficiency of the micronutrient cofactors (folates, vitamins B12 and B6), acts as a risk factor for a variety of developmental disorders [7]. Independently, Vit-B12 has a significant role in early neuronal and brain development [8] and folate deficiency is associated with neural tube defects (NTD) while errors of homocysteine metabolism promote a range of clinical complications [9]. Studies also show that exogenous supplementation of these nutrients to mothers during and prior to pregnancy helps reduce the risk of these disorders in the children [9]. Multiple investigations from different parts of the Indian subcontinent have confirmed, with minor differences, high prevalence of elevated Hcy, deficiency of Vit-B12 and folates along with strong genotype correlation with respect to Hcy levels in the population [10,11,12,13]. Further their association with different disorders has also been demonstrated [11,12]

The dismal state of the micronutrient (Vit-B12 and folate) levels related to Hcy-metabolic pathway in the general Indian population and their association with multiple disorders, raise concerns for the community at large, more specifically neonatal and child health. The premise of the present study is that if similar condition prevails in child at birth, it could be a risk factor for the high incidence of low birth weight and related complications in child health. We explore the status of these parameters in a group of just born children from the eastern region of India, and its influence, if any, on LBW and other health parameters. We report that the evaluated micronutrients in the children are even poorer than the adult population of the same region. More importantly, elevated Hcy, low vit-B12 and folates and *MTHFR677T*, all are independent risk factors against LBW. Unexpectedly, *MTHFR* 677T and low folate levels show association with neonatal hyperbilirubinemia (NNH), occurring in ~25% of the children, as independent risk factors. The results are presented in this paper.

## Materials and Methods

### Subjects

Cord blood samples (n=421) were collected from Division of Neonatology, Department of Pediatrics, Institute of Medical Sciences, Banaras Hindu University. In general, the babies belonged to lower middle economy group and were from rural area of eastern Uttar Pradesh, and western Bihar. The study was approved by Institutional ethical committee of Banaras Hindu University. Clinical information such as birth weight, foetal distress, sepsis, ABO incompatibility, G6PD deficiency, hypothyroidism, total serum bilirubin (TSB), hematological parameters, APGAR score etc were obtained from hospital data sheet. Children with less than 2.5kg birth weight were placed in the category of LBW. Hyperbilirubinemia in newborns was defined as peak TSB (total serum bilirubin) levels >95th percentile while control had physiological range of jaundice with peak TSB levels <75th percentile as per-hour-specific nomogram of the American Academy of Pediatrics [14] during the first 2 weeks of age. Exclusion criteria for the study were major congenital malformation and inability to attend follow-up clinic at 2 weeks of age. Both term (37-41 wk) and late preterm (34-36 wk) newborns were included in the study. A questionnaire regarding general food habits of parents (consumption frequency of milk, pulses (lentils), green leafy vegetables, fruits, egg and non-veg), medical history (hypertension, diabetics, cardiovascular, renal and gastro-intestinal issue), and B complex supplementation during gestation period, along with the written consent from all the parents.

As majority of the mothers came only during the period of delivery to the hospital and left after a few days, the follow up of subjects was not done, and since they were not regular visitors to the same hospital during pregnancy no systematic record of their metabolic status during pregnancy was maintained. The time of pregnancy was calculated mostly by LMP and rarely by clinical observation of the newborn by standard Ballard procedure. We did question them about intake of folate and Vit-B12 during pregnancy but most of them were unaware of the nature of “medicines” prescribed during pregnancy, and those 154 women who admitted to taking iron and folates were not confident of the duration and the period of intake during pregnancy. Vit-B12, it appears was not administered to any one, at least not on a sustained basis. As our objective was to investigate the relationship of newborn nutritional status, with potential clinical outcome, and not of the mother on which comprehensive data exist, we believe lack of maternal details in the studied cohort have minimal effect on the set objective of the present study.

### Collection of blood

The cord blood was collected in heparinised syringe. Plasma was separated immediately after sampling from 1ml of blood and stored at -80^°^ C until biochemical analysis of Hcy, B12 and folate was done. DNA was isolated from rest of the blood through salting out method and dissolved in T.E buffer (pH 8.0) and stored at 4^o^C.

### Biochemical Analysis of Metabolites

Homocysteine was measured by reverse phase HPLC for 421 plasma samples (Shimadzu, Kyoto, Japan) as previously described [13]. Vit-B12 and folate were measured for 334 randomly chosen samples (plasma was not sufficiently present for 87 samples) by chemiluminescence method (Immunolite1000, Siemens-Diagnostic-Products, and Flanders, NJ, USA) as per the manufacturer’s instructions.

### Gene Polymorphism analysis

Five gene polymorphisms in four genes of Folate/Hcy metabolic pathway were studied using gene-specific primers. Genomic DNA was subjected to PCR-RFLP for *SLC19A1* (*RFC1*) (G80A), *MTHFR* (C677T & A1298C), and *TCN2* (C776G), as earlier described [13,15] while *CBS* (844ins68 bp) was analysed by PCR with gene specific primers [13] and the presence or absence of 68bp insert was determined by running the PCR product on 2% agarose gel.

### Quality control

Reproducibility of biochemical measurements was ensured by performing intra assays where certain samples were measured in duplicates and also a few at two different time intervals. The measurement difference of <5% between the duplicates was accepted range for Hcy, Vit-B12 and folate values. The genotyping was repeated twice randomly for 20% of analysed samples, which yielded 100% identical results with the previous results.

### Statistical Analysis

For biochemical parameters like Hcy, Folate and Vit-B12, median and interquartile range was calculated. Comparison between two groups was done by non parametric test Mann–Whitney U-test, while for three groups One way ANOVA with Bonferroni correction was used. The allele frequencies of various studied SNP’s were tested for Hardy-Weinberg equilibrium and to assess the genotype and allele distributions among groups chi-square test was applied with Yates correction and p value, <0.05 was taken to be statistically significant. Binary Logistic regression analysis was done to find out potential risk factors for the dependent variables, LBW and NNH, by using SPSS version 16 statistical package (IBM, Armonk, NY, USA). Odds ratios (OR) were calculated, adjusted and given with 95% confidence interval. Cox and Snell R-square and Nagelkerke R-square were calculated to know the percentage of the clinical outcome (both in LBW and NNH) that could be explained with the total risk factors taken in to accountability for the study.

## Results

Cord blood samples of 421 neonates were examined. Of these, 16% were delivered prematurely (<37 weeks of gestation), 38% had low birth weight (<2.5kg) and 2 to 5% had other clinical features (table 1). A subgroup of the cohort (n=101; 24%) showed NNH as per guidelines of hour specific nomogram of American Academy of paediatrics and were subjected to phototherapy. All the 421 samples were assayed for serum Hcy. The median of Hcy was 11.7µmol/L, 25% being hypHcy (hypHcy≥ 15µmol/Lit). Unlike adults, in neonates there was no gender difference with respect to elevated levels of Hcy. From within this group of children, 334 randomly picked up samples were used for measuring folate and B12 levels While the median was 201 pg/ml for B12 and 3.6 ng/ml for folate, the most striking observation was the large proportion of neonates being deficient in vit.B12 (65%) and folate (27%) and hypHcy (25%). This frequency was far in excess of those observed in other parts of the world (table 2). The homocysteine levels among the newborns and adult population did not differ significantly while B12 and folate levels in newborns were significantly lower than in adults (table 3).

**Table 1:** Clinical profile of the cohort (n = 421).

Prematurity (<37 weeks)	(16%)
Low Birth Weight	(38%)
Gender (% male)	(56%)
Breastfeeding	(99%)
Excessive weight loss (≥ 10%)	(4.5%)
Foetal Distress	(3.0%)
Sepsis	(5.0%)
Rh-Incompatibility	(3.3%)
ABO-Incompatibility	(11.2%)
G6PD deficiency	(0.5%)
Hypothroidism	(2.6%)
TSB ≥ 95 percentile	(24%)

**Table 2:** Hcy, B12 and Folate levels in Neonates worldwide.

**Population**	**Homocysteine (n)**	**Vit-B12 (n)**	**Folate (n)**	**Reference**
**Brazilian**	7.4µmol/L (75)	579 pg/ml (143)	7.8 ng/ml (143)	[16]
**Brazilian**	6.6µmol/L (69)	347 pg/ml (69)	13 ng/ml (69)	[17]
**Norwegian**	6.2µmol/L (361)	404 pg/ml (361)	21.4 ng/ml (361)	[18]
**Chinese**	−	240 pg/ml (99)	7.3 ng/ml (99)	[19]
**Turkish**	7.5µmol/L (204)	236 pg/ml (204)	14.3 ng/ml (204)	[20]
**Turkish**	−	207 pg/ml (188)	17.8 ng/ml (188)	[21]
**Swiss**	7.8 µmol/L (123)	357 pg/ml (123)	16.6 ng/ml (123)	[22]
**Seychelles**	10.2µmol/L (135)	558 pg/ml (135)	18.7 ng/ml (135)	[23]
**Indian**	**11.7µmol/L (421)**	**201 pg/ml (334)**	**3.6 ng/ml (334)**	**Present study**

All values of B12 and Folate are converted into pg/ml and ng/ml respectively

**Table 3:** Frequency of minor alleles and Hcy, B12 folate levels in new born and adults.

**Genotype**	***MTHFR* C677T**	***MTHFR* A1298C**	***CBS* 844 ins 68bp**	***SLC19A1* G80A**	***TCN2* C776G**	**Hcy Median & IQR (µmol/L)**	**Vit-B12 Median & IQR (pg/ml)**	**Folate Median & IQR (ng/ml)**
**N1=421**	(T=0.15) **χ** ^2^ = 1.24 & p = 0.24	(C=0.27) **χ** ^2^ = 1.16 & p = 0.29	(I=0.04) **χ** ^2^ = 0.57 & p = 0.73	(A=0.42) **χ** ^2^ = 1.69 & p = 0.22	(G=0.65) **χ** ^2^ = 0.16 & p = 0.75	11.7 (8.3-14.7)	201 (159-248)*	3.6 (2.8-5.7)*
**N2=1426**	(T=0.12) **χ** ^2^ = 2.35 & p = 0.10	(C=0.28) **χ** ^2^ = 0.90 & p = 0.34	(I-0.03) **χ** ^2^ = 0.68 & p = 0.55	(A=0.42) **χ** ^2^ = 1.91& p = 0.16	(G=0.65) **χ** ^2^ = 0.23 & p = 0.64	12.1 (8.8-15.4)	**222 (178–273)#**	**4.9 (3.7–6.0)#**

N1 = New born, N2 = General population [13] , * = 334 & # = 1290 samples, p-value = 0.003 for Vit-B12, p-value ≤0.0001 for folate

### Genotypes of the Hcy-pathway genes and their association with Hcy, Vit-B12 and folate levels

Genotypes of 4 SNPs (*C677T and A1298C* in *MTHFR, C776G for TCN2, G80A for RFC1*) and 1 insertion (*844ins68bp for CBS*) were in Hardy-Weinberg equilibrium (table 3), Hcy levels were significantly higher in individuals having 677T allele (15.2µmol/L (CT), 19.1 µmol/L (TT) vs 10.8 µmol/L (CC)) of *MTHFR* and G80 allele of *RFC1*(11.8 µmol/L (GG), 12.6 µmol/L (GA) VS 9.9 µmol/L (AA), table 4). In *CBS* there were no homozygotes for 844ins68bp and heterozygotes also were only 30 out of 421 but their Hcy level was distinctly higher than in the homozygotes for the major allele. In contrast, *SLC19A1*80AA homozygote was apparently a protective genotype against elevated Hcy levels because the median Hcy level in 80 AA was significantly lower than in 80GG and 80GA, even in presence of T allele of *MTHFR* C677T the *SLC19A1*80AA (14.5 µmol/L) showed low levels of Hcy as compared with 80GA (16.3), 80GG (15.7) genotypes. MTHFR 1298C allele, though not statistically significant, showed some association with elevated levels of Hcy. The TCN2 C776G bore no significant impact on Hcy levels. MTHFR 677T allele and MTHFR 1298CC genotype were associated with low folate levels while other polymorphisms did not show any association with Vit-B12 and Folate levels. In the individuals genetically predisposed to elevated levels of Hcy, deficiency of Vit-B12 and/or folate led to further rise in Hcy levels, implying that the genotypic effect on Hcy levels was modulated by micronutrient levels. The study further showed a close association of micronutrient levels, Hcy levels and certain genotype with respect to clinical outcome of a new born.

**Table 4:** Interaction of SNP’s with folate / Hcy biomarkers:.

**Genotype**	**Median (Hcy µmol/L)**	**Median (B12 pg/ml)**	**Median (Folate ng/ml)**
*MTHFR* 677 **CC**	10.8	210	3.9
*MTHFR* 677 **CT**	15.2	172	3.3
*MTHFR* 677 **TT**	19.1	198	3.1
**P-value** One way ANOVA	<0.0001*	**0.32**	0.0008*
*MTHFR* 1298 **AA**	10.2	211	3.7
*MTHFR* 1298 **AC**	10.6	201	3.5
*MTHFR* 1298 **CC**	13.2	187	3.1
**P-value** One way ANOVA	0.23	0.13	**0.02**
*CBS* 844 ins68bp**WW**	11.6	214	3.7
*CBS* 844 ins68bp**WI**	12.2	186	3.6
**P-value** One way ANOVA	0.37	0.46	0.68
*SLC19A1* 80 **GG**	12.6	198	3.5
*SLC19A1* 80 **GA**	12.2	202	3.6
*SLC19A1* 80 **AA**	10.0	214	3.6
**P-value** One way ANOVA	0.0002*	0.32	0.63
*TCN2* 776 **CC**	12.6	198	3.2
*TCN2* 776 **CG**	11.9	201	3.6
*TCN2* 776 **GG**	11.2	212	3.6
**P-value** One way ANOVA	0.15	0.61	0.23

* indicates significant after Bonferroni Multiple comparison test

### Genetic and nutritional variants in Low Birth Weight

Nearly 40% of neonates in the studied cohort clinically belonged to low birthweight (LBW) category. The proportion of LBW children with elevated levels of Hcy, deficiency of Vit-B12 and folate was greater than those with normal weight (NW- ≥2500gm). When subjected to Mann-Whitney test, the difference was statistically significant for all the three parameters (table 5) However, when subjected to Bonferroni correction for all three, only low Vit-B12 levels showed significant association with LBW. For evaluating genotypic association with LBW, all the genotypes except CBS (in which no homozygotes of the variable allele were obtained), were tested by taking dominant model into consideration since heterozygous as well as homozygous variable genotypes tended to show association with LBW. The analysis showed T allele of *MTHFR* C677T and CC genotype of TCN2 significantly associated with LBW as risk factors, while association of rest of the SNP’s was statistically not significant (table 5). The significance of association of all the tested variables with LBW was further checked through Binary logistic multivariate regression analysis, taking other known clinical factors for LBW into consideration. Hyperhomocystenemia, Vit-B12 & folate deficiency along with T allele of MTHFR C677T were significantly associated with LBW, while G allele of TCN2 C776G turned out as protective against LBW. Rest of the SNP’s did not show association with LBW. This analysis also revealed high association of premature delivery with LBW (OR 16), and that the LBW children were predisposed to excessive weight loss (table 6). The Cox and Snell R-square model explains the occurrence of LBW with the chosen variables to an extent of 38% while the remaining is to be contributed by other etiological factors.

**Table 5:** Hcy/Folate pathway associated risk factors with LBW in newborn.

**Group**	**Hcy (µmol/L) Mann-Whitney test**	**Vit-B12 (pg/ml) Mann-Whitney test**	**Folate (ng/ml) Mann-Whitney test**	**MTHFR 677T Dominant model**	**TCN2 776G Dominant model**
LBW(160)	12.4	189	3.3	χ^2^ = 4.52^$^, **OR (95% CI)** = 1.6 (1.2–2.5)	χ^2^ = 7.26^$^, **OR (95% CI)** = 2.3 (1.3–4.1)
NW (261)	10.6	208	3.9		
**p-value**	**0.023**	0.016*	**0.032**	**0.038**	**0.009**

Hcy, B12, Folate median values, n=334 for B12 and Folate (n=138 for LBW and n=196 for NW), * = p-value significant after Bonferroni correction, $ = Chi-square value significant after Yates Correction.

**Table 6:** Binary logistic regression analysis for the risk factors associated with LBW.

Characteristics (n=334)*	**DF**	**P value**	**Adjusted Odds**	**95% C.I. for Odds**
**hypHcy**	1	**0.010**	**2.68**	**1.36-5.70**
Gender (male)	1	0.559	1.17	0.68-2.02
**Vit-B12 deficiency**	1	**0.037**	**2.41**	**1.34-4.50**
**Folate deficiency**	1	**0.045**	**2.40**	**1.31-5.16**
**MTHFR 677T**	1	**0.014**	**2.61**	**1.31-4.18**
MTHFR 1298C	1	0.161	1.28	0.65-3.33
CBS 844ins68bp	1	0.356	1.61	0.58-4.42
SLC19A1 80 G	1	0.632	0.85	0.43-1.67
**TCN2 776G**	1	**0.010**	**0.65**	**0.28-0.94**
**Excessive weight loss (≥10%)**	1	**0.010**	**1.60**	**1.32-2.91**
ABO incompatibility	1	0.270	1.60	0.69-3.72
**Prematurity (<37 weeks)**	1	**0.000**	**15.82**	**4.32-57.86**
Sepsis	1	0.240	1.92	0.64-5.71
Foetal distress	1	0.716	0.70	0.30-4.67
Dependent variable (LBW)	1	0.967	-	-

**Cox and Snell R square value = 0.39, Nagelkerke R square value = 0.52**

* N= 138 for LBW and N= 196 for controls

### Genetic and nutritional variants in Neonatal Jaundice

NNH is a clinical condition common in early neonatal life. In accordance with the hour-specific nomogram of the American Academy of Paediatrics, 101 children within the studied cohort fell under NNH (>95 percentile TSB level), 304 in the normal (<75 percentile TSB levels) categories, and 16 were excluded from either category as they fell between the <75 and >95 percentiles. When subjected to diverse clinical parameters such as prematurity, gender (64% males), excessive weight loss, foetal distress, sepsis and ABO incompatibility, all of them showed association with NNH to variable degrees.

Since more than 100 newborns developed NNH, we retabulated our data as a case-control study between the NNH and unaffected children to test if any of the variables studied had a predisposing effect on NNH. While the case children developed NNH within 72 hours of birth, controls were those who did not develop NNH up to a week after birth. There was no mandatory follow up of the “control” children after 7 days. T allele frequency of C677T in the NNH cases (n=101; 0.24) was twice that in controls (n=304; 0.12), while other SNPs did not vary among case and control groups thereby showing no significant association with clinical outcome. Similarly, proportion of individuals with deficiency of Vit-B12 as well as folate and elevated levels of Hcy in the cases was greater than in controls, and the difference was statistically significant (table 7) but up on Bonferroni correction only folate deficiency is turning out to be significantly associated with NNH. Genotyping analysis showed MTHFR 677T allele to be the only significant risk factor for NNH while rest of the studied SNP’s did not show any association. However, multiple regression analysis showed male gender, sepsis, ABO incompatibility, T allele of *MTHFR* C677T, and folate deficiency all independently associated with NNH while Vit-B12 and hypHcy did not qualify as independent risk factors (table 8). The Cox and Snell R-square model explains the occurrence of NNH with the chosen variables to an extent of 32% while the remaining is to be contributed by other etiological factors.

**Table 7:** Hcy/Folate pathway associated risk factors with NNH in newborn.

**Group**	**Hcy (µmol/L) Mann-Whitney test**	**Vit-B12 (pg/ml) Mann-Whitney test**	**Folate (ng/ml) Mann-Whitney test**	**MTHFR677T Dominant model**
NNH (101)	13.2	190	2.9	χ^2^ = 25.16^$^, **OR (95% CI)** = 3.3 (2.1-5.2)
Control (304)	11.3	207	3.9	
**p-value**	**0.023**	**0.038**	0.003*	**>0.0001**

Hcy, B12, folate median value, n=334 for B12 and Folate (n= 101 for NNH, n = 233 for Control) * = p-value significant after Bonferroni correction, $ = Chi-square value significant after Yates Correction.

**Table 8:** Binary logistic regression analysis for the risk factors associated with NNH.

**Characteristics (n=318)**	**DF**	**P value**	**Adjusted Odds**	**95% C.I. for Odds**
**Gender (male)**	**1**	**0.034**	**2.19**	**1.06–4.54**
hypHcy	1	0.238	1.95	0.80–3.73
Vit-B12 deficiency	1	0.336	1.79	0.65–3.20
**Folate deficiency**	**1**	**0.003**	**3.08**	**1.48–6.41**
**MTHFR 677T**	**1**	**0.002**	**3.92**	**1.63–9.42**
MTHFR 1298C	1	0.211	1.57	0.77–3.23
CBS 844ins68bp	1	0.508	1.64	0.37–7.11
SLC19A1 80A	1	0.657	1.36	0.51–2.74
TCN2 776G	1	0.580	0.95	0.37–2.05
Low Birth Weight	1	0.091	1.66	0.80–2.42
Excessive weight loss (≥10%)	1	0.198	1.39	0.45–2.02
**ABO incompatibility**	**1**	**0.000**	**6.70**	**2.45–18.32**
Prematurity (<37 weeks)	1	0.117	1.56	0.73–2.71
**Sepsis**	**1**	**0.000**	**13.80**	**4.52–42.11**
Foetal distress	1	0.559	2.11	0.17–25.97
Dependent Variable (NNH)		0.996	-	-

**Cox and Snell R square value = 0.33, Nagelkerke R square value = 0.42**

n=318 (Cases = 101, Control =217)

## Discussion

The present study originates from the fact that there is very high incidence of low birth weight cases in India, and that there is deficiency of Vit-B12 and folates and high level of Hcy in a large proportion of the adult population in the eastern region of India [9]. The rationale of the present study is that the parameters of the homocysteine metabolic pathway could be risk factors for poor gestational growth causing LBW and even child mortality. Briefly stated, ~25% of the cohort is hyperhomocysteinemic (28% in adults), 65% (adult 49%) is Vit-B12 and 27% (adult 11%) folate deficient, and genotypically frequency of *MTHFR* 677T and *SLC19A1* 80G is higher as risk factors for elevated Hcy levels, a trend similar to that of the adult population, only more severe. An earlier study [24] on a cohort of over 2000 children in age group of 6-30 months from northern India documents 48% children to be Vit-B12 and 32% to be folate deficient. Evidently, this pattern is wide-spread in India. When put in context of the global scenario on neonates, the group from India is by far the most impoverished in Vit-B12 and folate and highest in Hcy levels, all indicators of poor health status (table 2). Several studies on young toddlers from diverse parts of the world have conclusively shown that the neonatal status of these metabolites is retained until later in age, and is a reflection of the maternal environment during pregnancy [4,18]. In view of the fact that in the present study also the Vit-B12 and folate indices in newborns are considerably lower than in adults, we suspect that their mothers may have also had low levels of folate and vit-B12 and elevated Hcy levels. The other factor to modulate the neonatal hypHcy must be the genotype.

### Low birth weight and Impaired Folate/Hcy Metabolism

The focus of our study, however, is to explore possible association of the Hcy-pathway variables with LBW. About 38% (160/421) of the presently studied just-borns have LBW which is in accordance with the projected frequency of LBW (40%) in India [4]. Beside the previously known factors (Prematurity, loss of weight), elevated Hcy, low folate and vit-B12 levels and higher frequency of the T allele of MTHFR C677T all tend to be risk factors for LBW to various degrees (tables 5 & 6). There are several studies from different parts of the world, including those from India, which show that elevated Hcy and lowered Vit-B12 and folate in mothers during pregnancy are associated with low birth weight but the same studies fail to show similar association with the cord blood of the child [4,18]. In fact, in a number of these studies the association is in the negative order: heavier children have lower micronutrients and elevated Hcy than those with lower birth weight [18,25]. Seemingly our results are in disagreement with these results. However, it is important to understand that in most of the previous studies referred here the “low” Vit-B12 or folate in the child is not lower than their internationally accepted optimum levels, nor is the “low birthweight” a potential clinical outcome; it is only the lower range of the weight spectrum. In contrast, in the present study low birth weight is a potential clinical state (<2.5kg) and the low nutrients are indeed lower than the optimum, just as Hcy is much higher than the higher limit of the normal Hcy levels. It is also rational to expect that vit-B12 and folate deficiency and hypHcy (25%) in newborns not only indicate low availability of micronutrients during development but also might cumulatively impede foetal development and growth.

Therefore while maternal environment per se, is the major environmental contributor to the foetal health including the weight, if the nutrient levels dip too low in the child, it may add to the risk of lowering of the birth weight which may have health consequences in later part of life. In addition, the genotype *MTHFR* 677T is a risk factor while *TCN* 776G, which is the major allele in this population, could be protective against LBW.

### NNH and Folate/Hcy metabolic pathway

It is intriguing that neonatal jaundice, which is transient in nature, also shows statistically significant association with *MTHFR* 677T, elevated homocysteine and Vit-B12 and folate deficiency. However, following Bonferrroni correction and the binary logistic regression analysis, it turns out that only *MTHFR* 677T and folate deficiency are independent risk factors. This is the first study to show an association of MTHFR677T/folate deficiency with the development of NNH. Interaction of T allele of *MTHFR* C677T with low folate levels has been reported in studies on Norwegian [26] and Czech populations [27], which suggests that *MTHFR* 677T and folate deficiency, instead of being two independent factors, are causally related with the mutation contributing to the low level of folates. The same could be the situation in present study.

Folate and Vit-B12 (stored in liver) are crucial for maturation and proliferation of RBC. Their deficiency causes failure of RBC maturation leading to rapid lysis [28]. This results in excess haem degradation and additional production of bilirubin, which is to be metabolized by liver. Since, in neonates the functional capacity of liver is only about 1% of the adult liver [29], we suspect that excess bilirubin due to RBC lysis and low functional ability of liver would result in inefficient clearance of bilirubin, causing its accumulation and NNH. Additionally, hypomethylation due to MTHFR 677T and folate deficiency, which has a global influence on gene expression could also be a factor leading to predisposition for NNH, as much as for LBW or most other developmental disorders. These speculations need to be functionally established. Regardless of the possible mechanism(s), it is obvious from this study that the 1-Carbon metabolism pathway constituents are risk factors of LBW and NNH. By implication, maintenance of a better gestational regime of the mother could substantially alleviate the possibility of LBW and NNH.

In summary, this work reemphasises the need of antenatal care which will not only improve maternal health but also lessen neonatal disease burden. It is important that those responsible for overseeing community health in countries like India should consider fortification with necessary micronutrients for mothers-to-be and neonates.
