# Correction: Low Birthweight (LBW) and Neonatal Hyperbilirubinemia (NNH) in an Indian Cohort: Association of Homocysteine, Its Metabolic Pathway Genes and Micronutrients as Risk Factors

**DOI:** 10.1371/annotation/9efa112d-83ed-40ef-a99e-d691a614880d

**Published:** 2013-09-20

**Authors:** Krishna Kishore Sukla, Pankaj Kumar Tiwari, Ashok Kumar, Rajiva Raman

Affiliations numbers 1 and 2 for the first and fourth authors were given incorrectly.

The correct citations are: 1, Cytogenetics Laboratory, Department of Zoology, Banaras Hindu University, Varanasi, Uttar Pradesh, India; 2, Centre for Genetic Disorders, Banaras Hindu University, Varanasi, Uttar Pradesh, India. 

